# Emerging quantitative CCTA imaging biomarkers for cardiovascular risk stratification: a narrative review

**DOI:** 10.3389/fcvm.2026.1850945

**Published:** 2026-08-04

**Authors:** Ruben Mora, Kyvan Irannejad, Nia Abbas, Phanidhar Mogga, Beshoy Iskander, Logan Hubbard, Sion Roy, Suvasini Lakshmanan, Matthew Budoff, Srikanth Krishnan

**Affiliations:** 1 Harbor-UCLA Medical Center and The Lundquist Institute for Biomedical Innovation at Harbor-UCLA, Torrance, CA, United States; 2 Harbor–UCLA Medical Center, Torrance, CA, United States; 3 UCLA Radiology, Los Angeles, CA, United States

**Keywords:** cardiac CT angiography, cardiometabolic risk, epicardial adipose tissue, FFR-CT, hepatic steatosis, imaging biomarkers, pericoronary fat attenuation index, quantitative plaque

## Abstract

**Background:**

Cardiac computed tomography angiography (CCTA) has evolved beyond anatomical stenosis assessment into a comprehensive platform for cardiovascular and cardiometabolic risk stratification. Advances in postprocessing and artificial intelligence now enable automated quantification of multiple imaging biomarkers from a single acquisition, including coronary plaque characteristics, CT-derived fractional flow reserve (FFR-CT), epicardial adipose tissue (EAT), pericoronary adipose tissue (PCAT), and hepatic steatosis.

**Purpose:**

In this narrative review, we synthesize current imaging biomarkers, evaluate their individual and combined prognostic value, and propose a conceptual multimarker framework for cardiovascular risk stratification—recognizing that several domains remain investigational and are not yet ready for routine, biomarker-guided management.

**Key findings:**

Quantitative plaque analysis identifies high-risk features — including low-attenuation plaque, positive remodeling, and napkin-ring sign — that independently predict major adverse cardiovascular events (MACE) beyond stenosis severity. FFR-CT carries a Class 2a guideline recommendation for intermediate lesions and demonstrates superior vessel-level diagnostic accuracy compared with SPECT and comparable performance to PET in head-to-head trials. EAT volume and density independently predict incident coronary heart disease, atrial fibrillation, and all-cause mortality across large prospective cohorts. Pericoronary fat attenuation index (FAI) reflects local coronary inflammation, independently predicts MACE after adjustment for conventional risk factors and coronary calcium, and decreases in response to high-dose statin therapy. Hepatic steatosis, identifiable from the same noncontrast acquisition used for calcium scoring, is associated with a 64% increased odds of cardiovascular events in a meta-analysis exceeding 34,000 adults and predicts both plaque progression and high-risk plaque features in longitudinal registries. Emerging multimarker models combining these domains demonstrate incremental discriminatory value beyond individual imaging biomarkers or traditional clinical risk scores.

**Conclusion:**

CCTA provides a pragmatic, multidimensional framework that integrates anatomic, functional, inflammatory, and metabolic information from a single noninvasive examination. While standardization, longitudinal validation, and equitable representation in research cohorts remain unresolved challenges, ongoing advances in AI-driven image analysis and multiomics integration may, if validated in prospective outcome studies, support the future translation of quantitative CCTA imaging biomarkers into more personalized cardiovascular care.

## Introduction

Cardiovascular disease remains the leading cause of death globally, despite advances in diagnostic and therapeutic strategies (1). Noninvasive imaging is central to risk stratification, and cardiac computed tomography angiography (CCTA) is now a cornerstone modality for anatomical assessment of the coronary arteries. Major clinical guidelines strongly endorse CCTA, granting it a Class I, Level of Evidence A recommendation across two pivotal documents: the 2024 ESC Guidelines on chronic coronary syndromes (CCS), for patients with suspected CCS and low-to-moderate (>5%–50%) pretest likelihood of obstructive coronary artery disease (CAD) to establish diagnosis and assess MACE risk, and the 2021 AHA/ACC/ASE/CHEST/SAEM/SCCT/SCMR Guideline for the Evaluation and Diagnosis of Chest Pain, for intermediate-risk patients with acute chest pain and no known CAD after a negative or inconclusive workup for acute coronary syndrome, where it aids in excluding atherosclerotic plaque and obstructive CAD (2, 3).

Traditionally, CCTA has been used primarily to identify or exclude obstructive coronary stenosis; however, stenosis severity alone incompletely characterizes atherosclerotic burden and cardiovascular risk, particularly in non-obstructive disease (4–6). Advances in scanner technology, imaging acquisition, and postprocessing have expanded the value of CCTA beyond stenosis assessment, enabling automated coronary segmentation and quantitative plaque mapping, including identification of high-risk features such as low attenuation, positive remodeling, and napkin-ring sign (7–9).

CCTA also offers functional assessment through FFR-CT, which provides lesion-specific physiological information by simulating coronary hemodynamics. In this way, FFR-CT helps bridge the gap between anatomical stenosis severity and physiological significance (10, 11).

Beyond the coronary lumen and vessel wall, routine cardiac CT can also provide extracoronary and metabolic imaging biomarkers that reflect the broader inflammatory and cardiometabolic milieu underlying atherosclerosis. Pericoronary adipose tissue (PCAT), also termed perivascular adipose tissue, is a dynamic indicator of vascular inflammation that has been linked to endothelial dysfunction and plaque vulnerability, while changes in CT attenuation, termed the fat attenuation index (FAI), may reflect the local inflammatory state (12–14). Epicardial adipose tissue (EAT), a metabolically active fat depot located between the myocardium and the visceral pericardium, is associated with coronary calcification and plaque burden, reflecting local paracrine inflammatory effects (15, 16). Cardiac CT scans can also identify hepatic steatosis and visceral adiposity, both of which are independent indicators of metabolic dysfunction and cardiovascular risk (17, 18).

Together, the integration of anatomical, plaque-based, functional, and extracoronary metabolic information within a single imaging modality represents an important step toward more comprehensive, patient-specific cardiovascular risk stratification.

Despite these innovations, several challenges limit widespread clinical adoption. Current guidelines continue to emphasize stenosis severity, while emerging quantitative imaging biomarkers, such as plaque composition, pericoronary inflammation, and hepatic fat, remain underutilized. Furthermore, issues of reproducibility, standardization, and longitudinal validation persist, emphasizing the need for integrated frameworks and evidence-based workflows.

This narrative review synthesizes recent progress in CCTA-based risk assessment, discusses the contribution of each imaging biomarker to cardiovascular risk prediction, appraises the supporting evidence, and proposes a conceptual model for their integration. We reviewed literature indexed in PubMed/MEDLINE through early 2026, prioritizing guideline and society consensus documents, large prospective cohorts, registries, meta-analyses, and pivotal diagnostic-accuracy studies; because this is a narrative synthesis rather than a systematic review, study selection was interpretive rather than exhaustive and reflects the authors' appraisal of representative and high-impact evidence. The biomarkers discussed differ substantially in biological meaning, validation maturity, reproducibility, and readiness for clinical use; throughout, we distinguish guideline-endorsed applications (quantitative plaque burden and FFR-CT) from investigational or opportunistic markers (EAT, PCAT/FAI, and hepatic steatosis), and we summarize their evidence status in Table 1.

**Table 1:** Six pathobiologic domains of the integrated CCTA multimarker framework: biomarker content and evidence status.

Domain	Pathobiologic signal	Evidence status
Plaque quantity (anatomic burden)	Total atherosclerotic burden; stenosis severity; segment involvement score	**Guideline-endorsed** (Class I, LOE A) (2, 3); SCCT consensus (26)
Plaque morphology (high-risk structural features)	LAP, NCP, positive remodeling, napkin-ring sign (HRP modifier)	**Society-endorsed** (2025 AHA scientific statement) (57)
FFR-CT (functional ischemia)	Lesion-specific hemodynamic significance; revascularization guidance	**Guideline-endorsed** (Class 2a) (3)
Epicardial adipose tissue (systemic inflammatory/ metabolic status)	EAT volume and density; paracrine inflammatory signaling; metabolic syndrome surrogate	*Investigational*; observational cohorts (Heinz Nixdorf, SCOT-HEART) (16, 71)
Pericoronary adipose tissue (local coronary inflammation)	Pericoronary FAI; coronary inflammation index	*Investigational*; observational cohorts (CRISP-CT) (13); no guideline threshold established
Liver adiposity (systemic metabolic risk)	Hepatic steatosis; insulin resistance surrogate; MASLD screen	*Investigational*; observational cohorts (Rotterdam, Framingham) (100, 101); no clinical threshold for CV risk modification

Guideline-endorsed = Class I or 2a recommendation in ACC/AHA or ESC formal guidelines, or core CAD-RADS 2.0 component. Society-endorsed = recognized and endorsed by major professional society scientific statement without formal Class/LOE grading.

AHA, American Heart Association; CAD-RADS, Coronary Artery Disease Reporting and Data System; CCTA, coronary computed tomography angiography; EAT, epicardial adipose tissue; ESC, European Society of Cardiology; FAI, fat attenuation index; FFR-CT, fractional flow reserve derived from coronary CT angiography; HRP, high-risk plaque; LAP, low-attenuation plaque; LOE, level of evidence; MASLD, metabolic dysfunction-associated steatotic liver disease; NCP, noncalcified plaque; PCAT, pericoronary adipose tissue; SCCT, Society of Cardiovascular Computed Tomography.

## Quantitative plaque analysis

### Methods of quantification

CCTA enables noninvasive quantification of coronary atherosclerosis from a single dataset. Total plaque volume is measured in cubic millimeters, and plaque composition is classified by Hounsfield units (HU): calcified (>350 HU), noncalcified (30–350 HU), and low-attenuation plaque (<30 HU) (19). Vessel geometry is further characterized by the remodeling index (ratio of lesion to reference cross-sectional area or diameter) (20).

**Figure 1:**
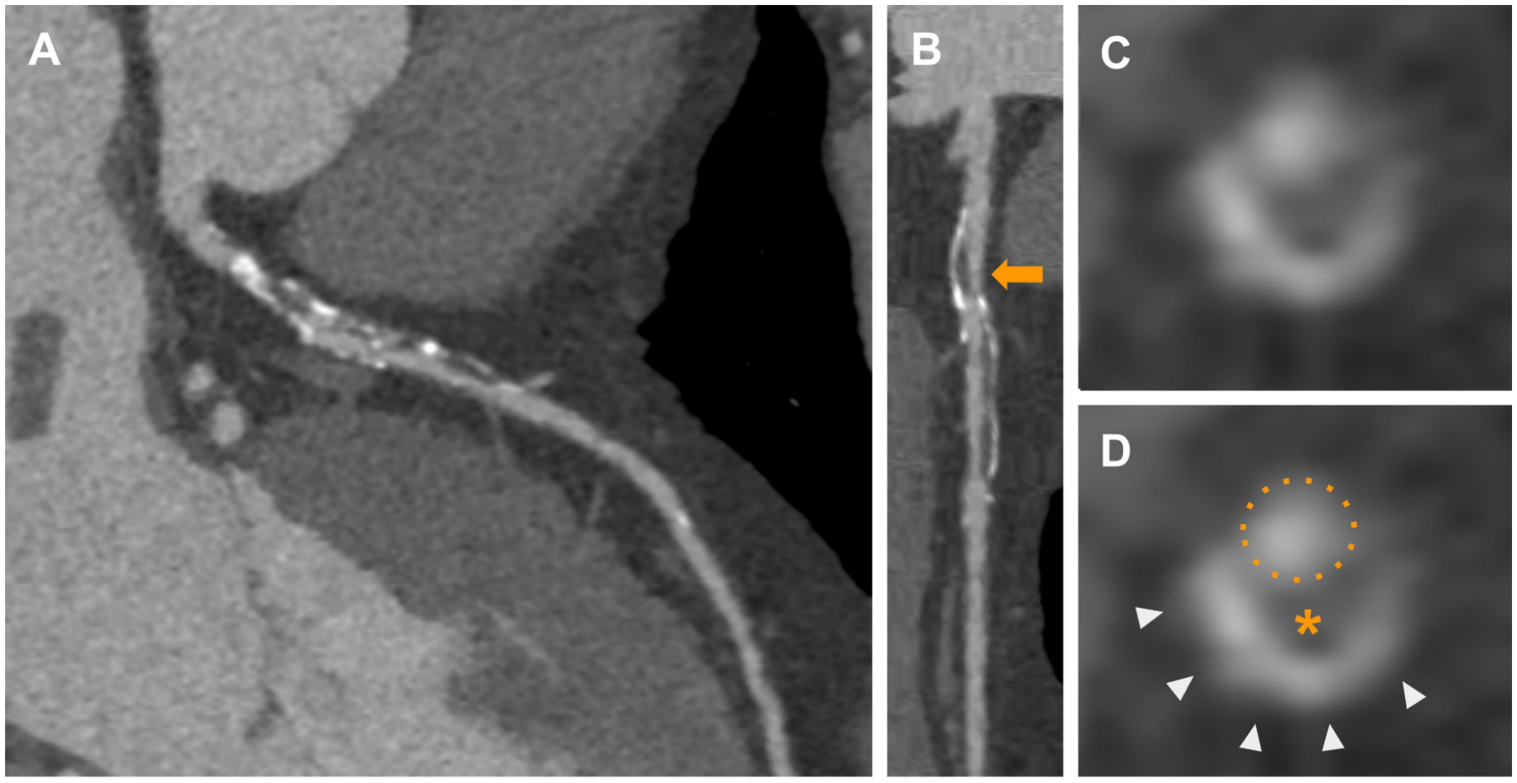
Severe stenotic lesion (orange arrows) of the proximal left anteriod descending (LAD) artery is demonstrated in the curved multiplanar **(A)** and stretched reconstruction view **(B)** A cross-sectional image perpendicular to the vessel lumen **(C** and **D)** shows an outer circumferential high-attenuation area (white arrowheads) and a central low-attenuation core (*). The dotted orange line outlines the lumen.

**Figure 2:**
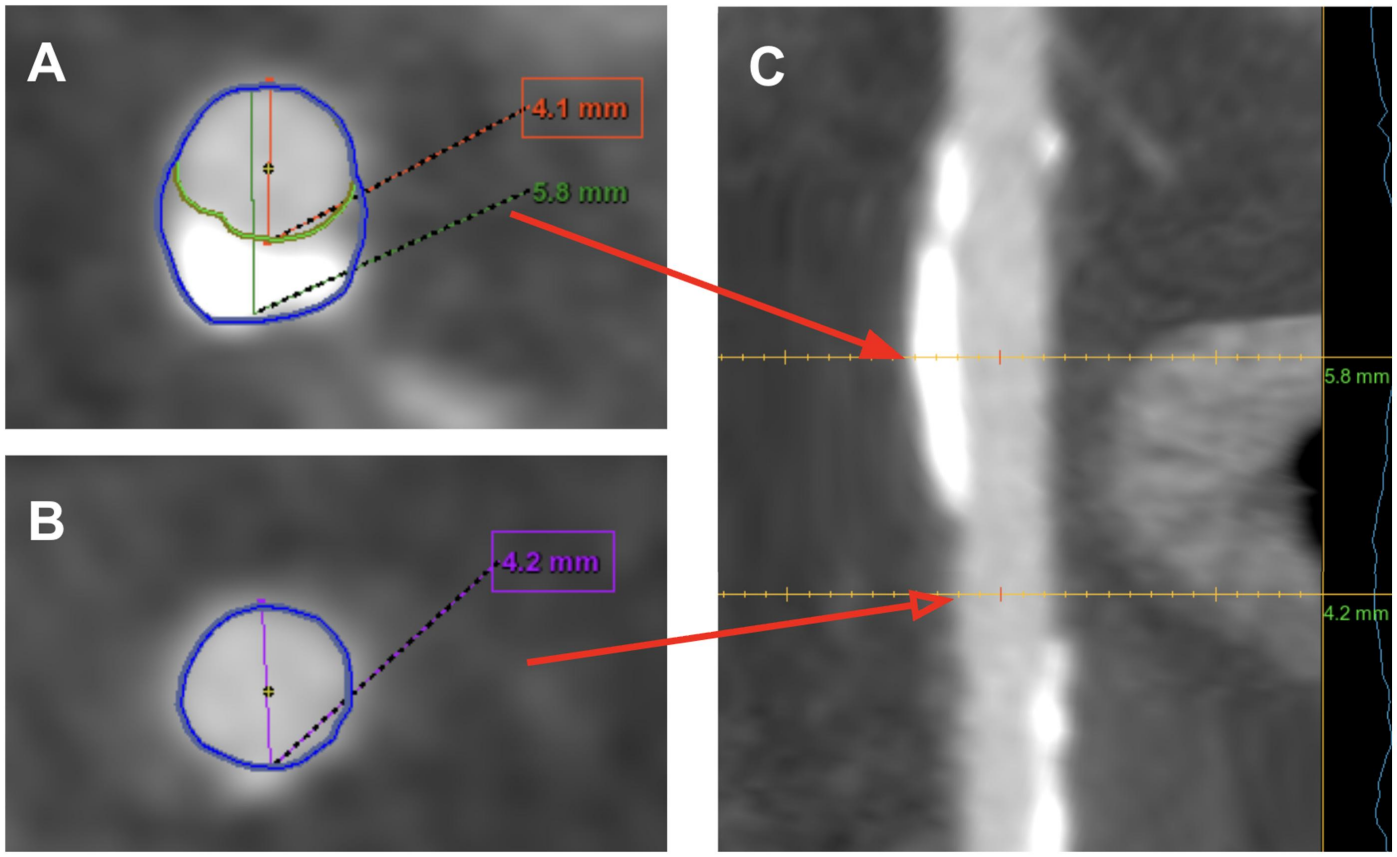
Panels **(A,B)** show cross-sections from the lesion (solid arrow) and reference (hollow arrow) sites depicted in panel **(C)** the reference vessel diameter is 4.2 mm, and the lesion's outer diameter is 5.8 mm, yielding a remodeling index of 1.38 (>1.10), consistent with positive remodeling.

**Figure 3:**
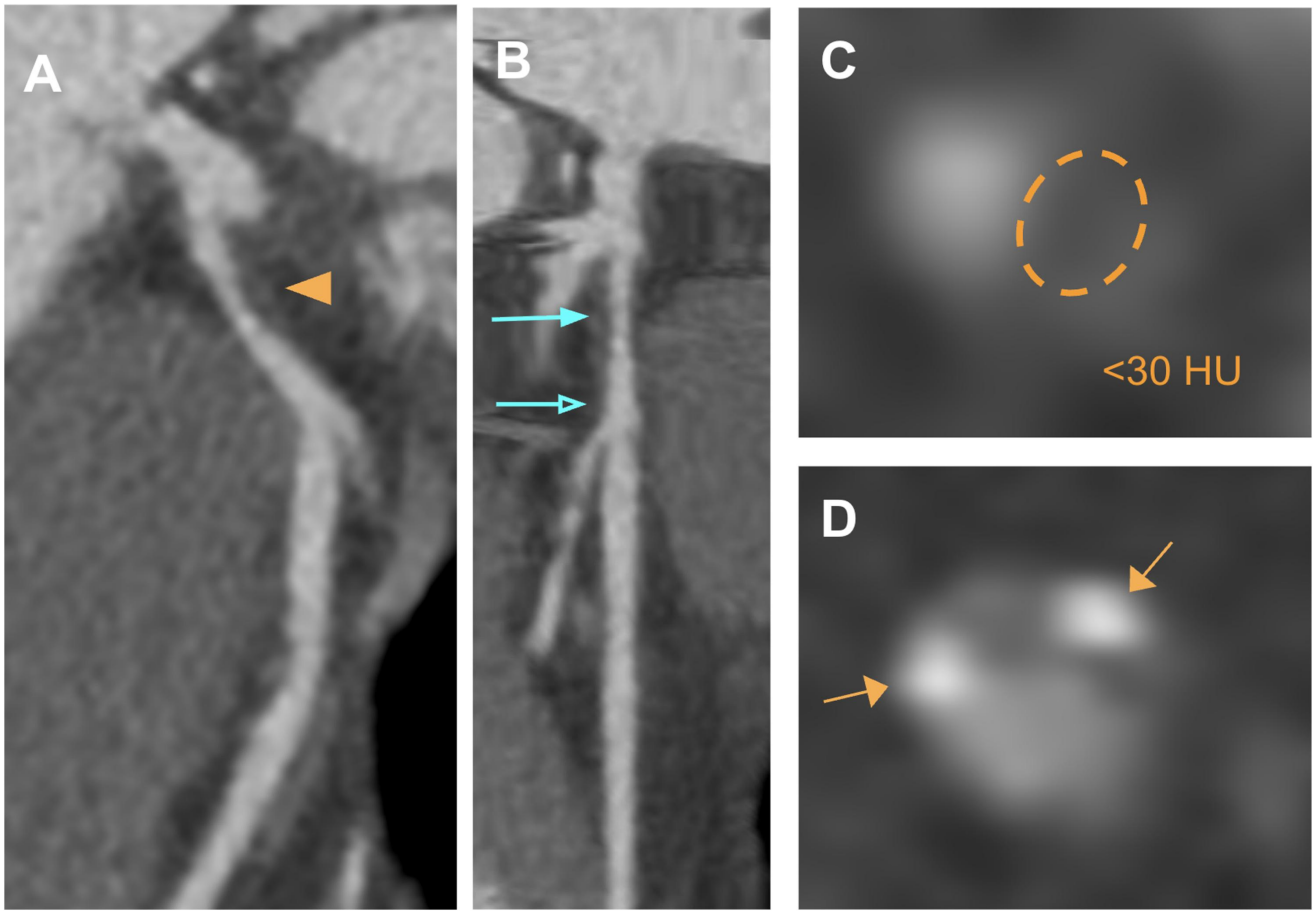
**(A)** the curved multiplanar view, with an orange arrowhead, and **(B)** the orthogonal cross-sectional view both highlight the presence of low attenuation plaque, defined by a density of less than 30 HU as seen in panel **(C)** the presence of spotty calcification is shown by orange arrows pointing to small, focal calcium deposits **(D)**

High-risk plaque features include the napkin-ring sign (Figure 1), positive remodeling (Figure 2), low attenuation core, and spotty calcification (Figure 3). These features predict future adverse cardiovascular events independent of stenosis severity (21, 22).

### Tools and techniques

**Figure 4:**
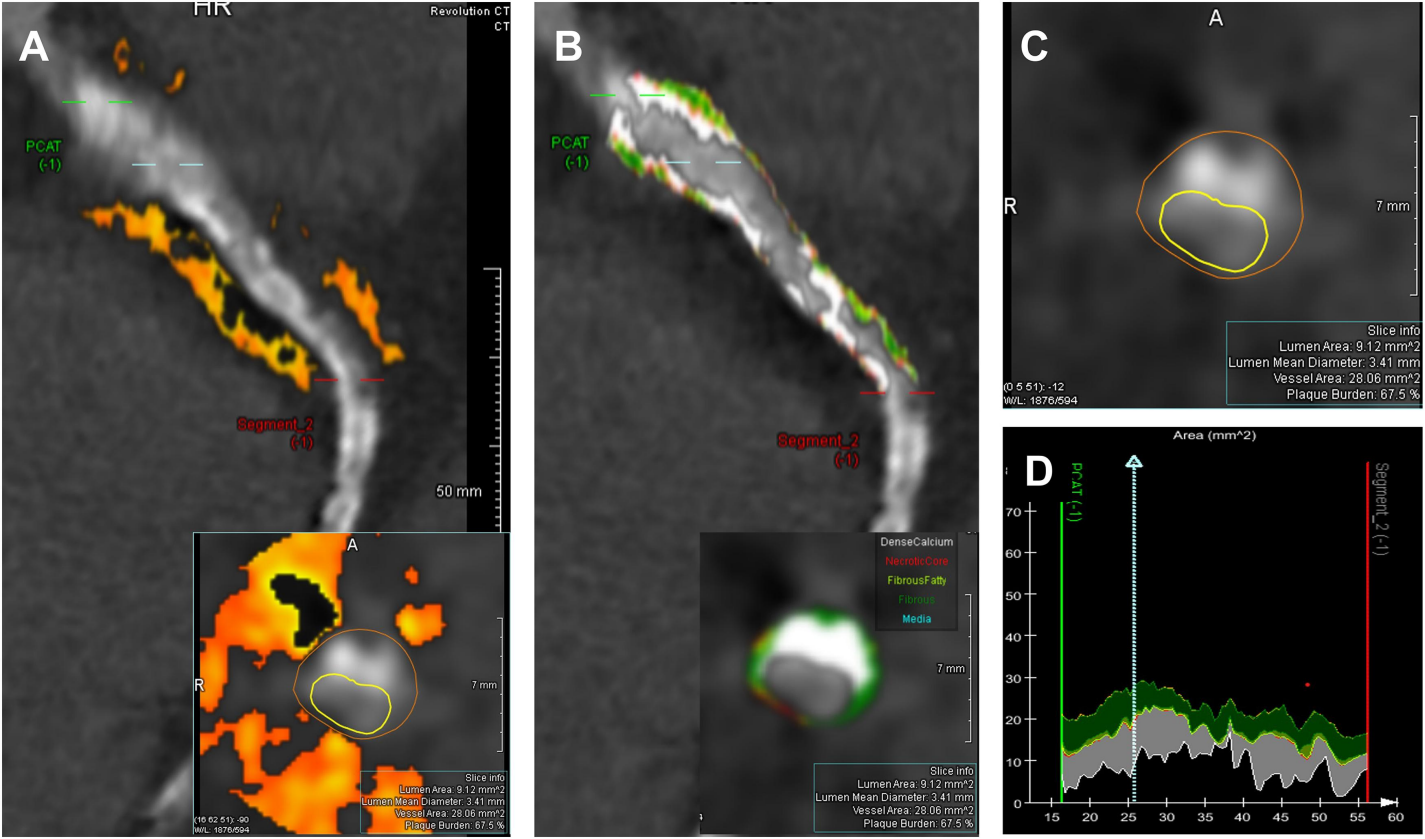
Quantitative imaging biomarkers derived from CCTA enhance cardiovascular risk assessment. **(A)** Multiplanar and orthogonal view for quantifying Pericoronary Adipose Tissue (PCAT) attenuation, a marker of local coronary inflammation. **(B)** Plaque characterization distinguishes calcified (>350 HU) from non-calcified (30–350 HU) components. **(C)** Automated contours of the lumen and vessel wall. **(D)** Histogram of the quantified plaque area. Together, these quantitative parameters offer a superior assessment of atherosclerotic burden and cardiovascular risk beyond traditional stenosis evaluation. Obtained on QAngio.

Semiautomated software platforms, including AutoPlaque, Syngo.Via Frontier and QAngio CT, facilitate plaque quantification. An example of a comprehensive quantitative workflow, including PCAT attenuation, plaque HU characterization, vessel wall modeling, and automated plaque histogram analysis, is shown in Figure 4. AI-enhanced platforms such as Cleerly Plaque Analysis, HeartFlow Plaque Analysis, and Elucid PlaqueID use machine learning to automate plaque segmentation and classification, reducing interobserver variability and analysis time (23–25). The SCCT has endorsed initiatives to standardize plaque classification metrics and improve cross-study comparability (26). A consolidated overview of currently available software platforms for each CCTA-derived biomarker, together with representative validation studies, is provided in Supplementary Table S1.

### Clinical implications

Low attenuation plaque (LAP) burden and noncalcified plaque (NCP) volume independently predict major adverse cardiac events (MACE) (27). Among quantitative plaque metrics, total plaque volume is one of the most robustly validated CCTA imaging biomarkers, with consistent prognostic value across large prospective cohorts and registries including SCOT-HEART and PARADIGM (27, 28). Total and percent atheroma volume independently predict MACE beyond stenosis severity and traditional risk scores, and AI-driven quantitative plaque assessment further refines this prediction in multicenter cohorts (23, 29). Together with low-attenuation and noncalcified plaque burden, total plaque volume now represents a foundational anatomic biomarker on which the integrated multimarker framework is built. Serial CCTA allows objective tracking of plaque progression or regression to assess treatment response, including lipid-lowering therapy (statins and nonstatin adjuncts) and newer cardiometabolic therapies such as glucagon-like peptide-1 (GLP-1) receptor agonists (30, 31). In the multicenter LOCATE study, progression of total and noncalcified plaque volume was attenuated by moderate-intensity and arrested by high-intensity lipid-lowering therapy (high-intensity statins or PCSK9 inhibitors), with regression of high-risk plaque features occurring more frequently under high-intensity treatment (32). Serial imaging can likewise track response to anti-inflammatory regimens: in patients with recent acute coronary syndrome, low-dose colchicine was associated with a significant reduction in low-attenuation plaque volume on serial CCTA, independent of statin intensification and low-density lipoprotein cholesterol lowering (33). Plaque composition analysis further informs titration of lipid-lowering or anti-inflammatory treatment. In the PARADIGM registry, lesions that subsequently caused acute coronary syndromes exhibited greater low-attenuation (fibrofatty and necrotic core) plaque volume and more high-risk features at baseline, together with more rapid progression, largely independent of stenosis severity, demonstrating the prognostic value of quantitative plaque phenotyping beyond luminal narrowing (34).

### Limitations and reproducibility

Technical limitations include variability in image acquisition parameters, such as tube voltage, contrast timing, and reconstruction kernel, which can affect plaque density assessment (35). Tube voltage similarly influences pericoronary adipose tissue attenuation and the derived FAI: because PCAT attenuation increases at higher kV, standardized tube-voltage protocols or published conversion factors are recommended when PCAT/FAI is compared longitudinally or across centers (36). Motion artifacts and partial volume effects may confound small plaque feature evaluation (37). Analytical challenges arise from interplatform differences in HU thresholds and segmentation algorithms, reducing reproducibility across studies (38). Clinically, the lack of standardized cutoffs for LAP and NCP burden limits their integration into management decision algorithms. Photon-counting CT (PCCT) has been shown to substantially affect coronary plaque volume assessment compared with conventional energy-integrating detector CT, with implications for both compositional thresholds and longitudinal follow-up.

## CT-Derived fractional flow reserve

### Principles and physics

Fractional flow reserve from CCTA (FFR-CT) integrates anatomical stenosis assessment with invasive physiology. It estimates lesion-specific ischemia directly from a standard CCTA acquisition, avoiding additional stress testing or pharmacologic hyperemia (39–41). Three methodological families are in use: computational fluid dynamics (CFD), plaque-based ischemia surrogates, and deep learning (42–46).

CFD approaches solve the Navier-Stokes equations on three-dimensional coronary reconstructions from CCTA. Typical steps include coronary tree segmentation, estimation of subtended myocardial mass, derivation of rest and hyperemic boundary conditions scaled to tissue demand, and CFD simulation to compute flow and pressure gradients across lesions (47, 48). HeartFlow FFR-CT (HeartFlow, Inc., Mountain View, CA) is the best-validated implementation, with multicenter diagnostic accuracy vs. invasive fractional flow reserve (FFR). Although computationally intensive, modern cloud infrastructure has made turnaround times practical for routine use (49–51).

Plaque-based surrogates infer ischemia potential from vessel geometry and plaque morphology without simulating hemodynamics. Continuous-value models, such as vascuCAP (Elucid, Inc., Boston, MA), use histology-linked feature extraction to generate FFR-like estimates (52, 53). Classification models, such as AI-QCT Ischemia (Cleerly, Inc., Denver, CO), categorize lesions as ischemic or nonischemic using quantitative plaque features (23, 54). These methods are fast and correlate with invasive FFR, though they do not reproduce the full continuous pressure gradient that CFD provides.

Deep learning models have been trained on large datasets that pair coronary anatomy with reference FFR values from CFD or invasive measurements. They predict per-lesion or along-the-vessel FFR directly from the images, offering rapid inference (45, 46). Performance depends on the quality of the training data, the labeling strategy, and generalizability across scanners and populations.

### Comparison to invasive FFR and stress testing

Invasive FFR remains the reference standard. FFR-CT shows strong concordance across multicenter studies. In a systematic review of 908 vessels, the diagnostic accuracy of FFR-CT vs. invasive FFR was 81.9% (55). Accuracy is highest at the extremes: FFR-CT <0.60 (86%) and >0.90 (98%), but falls sharply in the gray zone of 0.70–0.80 (≈ 46%). Reliable accuracy thresholds were observed at <0.63 or >0.83, with near-perfect performance at <0.53 or >0.93. Thus, FFR-CT provides high confidence when results are clearly abnormal or normal, although accuracy diminishes near the ischemic threshold.

Head-to-head comparisons from the PACIFIC trial showed that FFR-CT provides superior vessel-level diagnostic accuracy compared with single-photos emission computed tomography (SPECT) and even positron emission tomography (PET), provided CCTA images were analyzable (51). Detailed performance metrics at both the per-vessel and per-patient levels are summarized in Table 2. Briefly, FFR-CT achieved the highest vessel-level AUC (0.94 vs. 0.87 for PET and 0.70 for SPECT); at the per-patient level, PET demonstrated higher overall accuracy (88% vs. 78%), while AUC values for FFR-CT and PET were comparable (0.92 vs. 0.91) (11).

**Table 2:** Diagnostic performance of FFR-CT, SPECT, and PET versus invasive FFR: PACIFIC trial results.

Diagnostic performance	FFR-CT	SPECT	PET
**Per-vessel analysis**			
Accuracy, %	87	82	80
AUC	0.94	0.70	0.87
**Per-patient analysis**			
Accuracy, %	78	—	88
AUC	0.92	0.75	0.91

AUC, area under the curve; FFR-CT, fractional flow reserve derived from coronary CT angiography; PET, positron emission tomography; SPECT, single-photon emission computed tomography. Reference standard: invasive fractional flow reserve. Per-patient SPECT accuracy not reported separately in PACIFIC. FFR-CT analysis restricted to patients with analyzable CCTA images. Although PET demonstrated higher per-patient overall accuracy (88% vs. 78%), FFR-CT achieved a comparable AUC (0.92 vs. 0.91). These modalities assess complementary aspects of ischemic physiology and should not be considered interchangeable.

FFR-CT integrates anatomical definition with lesion-specific physiology, whereas SPECT and PET assess downstream perfusion and elements of microvascular function. These modalities offer complementary, rather than interchangeable, insights for evaluating and managing patients with suspected or established CAD (2, 56).

### Clinical utility

FFR-CT improves diagnostic precision in patients with intermediate coronary lesions (40%–70% diameter stenosis) by identifying physiologically significant stenoses that warrant revascularization, while avoiding unnecessary interventions for anatomically severe but nonischemic lesions (50). By deriving lesion-specific physiology from the index CCTA, FFR-CT can also reduce the need for additional downstream noninvasive ischemia testing, such as SPECT myocardial perfusion imaging or CT perfusion, thereby sparing patients further radiation exposure and testing. The integration of anatomical and functional data enhances decision-making, particularly in cases where conventional CCTA findings are equivocal. This functional integration is further supported by the 2025 AHA Scientific Statement on nonobstructive CAD, which endorses the CAD-RADS 2.0 framework, including FFR-CT as an ischemia modifier, and recognizes physiologic assessment as an important complement to plaque burden evaluation in risk stratification (57).

Abnormal FFR-CT values (≤0.80) are associated with higher rates of major adverse cardiovascular events (MACE) and revascularization. In the ADVANCE Registry, patients with FFR-CT ≤0.80 underwent revascularization more frequently and experienced higher event rates compared with those with values >0.80, emphasizing the prognostic value of CT-derived physiological assessment (58).

Clinical evidence from both the ADVANCE Registry and the PLATFORM trial demonstrates that FFR-CT–guided strategies reduce unnecessary invasive coronary angiography while maintaining safety and ensuring appropriate revascularization. The ADVANCE Registry highlighted the impact of FFR-CT on management decisions and outcomes in real-world practice (59). In contrast, the PLATFORM study showed that an FFR-CT–guided pathway in patients with planned invasive angiography was associated with equivalent clinical outcomes but lower rates of unnecessary catheterization and reduced costs at 1 year (60).

### Limitations

FFR-CT has important limitations, as motion artifact, inadequate contrast opacification, and extensive coronary calcification can reduce diagnostic accuracy and impair lumen segmentation and centerline tracking (61). Diagnostic accuracy is also notably reduced within the FFR-CT “gray zone” of 0.70–0.80, where reported per-vessel agreement with invasive FFR falls to approximately 46%. In this range, additional invasive physiologic assessment may be required before revascularization decisions, and clinicians should interpret borderline FFR-CT values with appropriate caution (55). CFD-based methods, although powerful, are computationally intensive and depend on simplifying assumptions, such as rigid vessel walls, laminar and Newtonian flow, absence of collaterals, and uniform pressures and resistances, that may not apply in patients with microvascular dysfunction, diabetes, left ventricular hypertrophy, or prior myocardial infarction. Evidence in higher-risk subgroups, including those with prior revascularization with stent or coronary artery bypass grafting (CABG), remains limited, and variability in reimbursement continues to pose barriers to broader clinical adoption (62). Ultra-high-resolution imaging enabled by PCCT may further influence FFR-CT diagnostic accuracy, particularly in heavily calcified or small-caliber vessels (63).

## Epicardial adipose tissue

### Anatomy and physiological role

EAT is a unique type of visceral fat located between the myocardium and the visceral pericardium. The absence of a fascial boundary allows shared microcirculation with the myocardium and coronary arteries, enabling paracrine and vasocrine signaling (64).

Under physiological conditions, EAT confers cardioprotection by providing mechanical support, supplying free fatty acids to the adjacent myocardium, and secreting protective adipokines with anti-inflammatory and anti-atherogenic effects (64, 65). With metabolic stress such as obesity or diabetes, EAT becomes inflamed and dysfunctional, transitioning toward a brown-to-white phenotype (66). At birth, brown tissue is more common, but with age, it transitions to a more inflammatory “white” state, a process accelerated by metabolic stress. This tissue experiences hypertrophy and increased lipid accumulation, secreting inflammatory cytokines such as interleukin-6 (IL-6), monocyte chemoattractant protein-1 (MCP-1), and tumor necrosis factor-a (TNF-α), and is infiltrated by M1 macrophages. These changes result in elevated reactive oxygen species, fibrosis, and additional adipose tissue remodeling. CCTA can quantify these changes by measuring tissue density in HU (65, 67).

#### Quantification methods: volume and attenuation in CCTA

**Figure 5:**
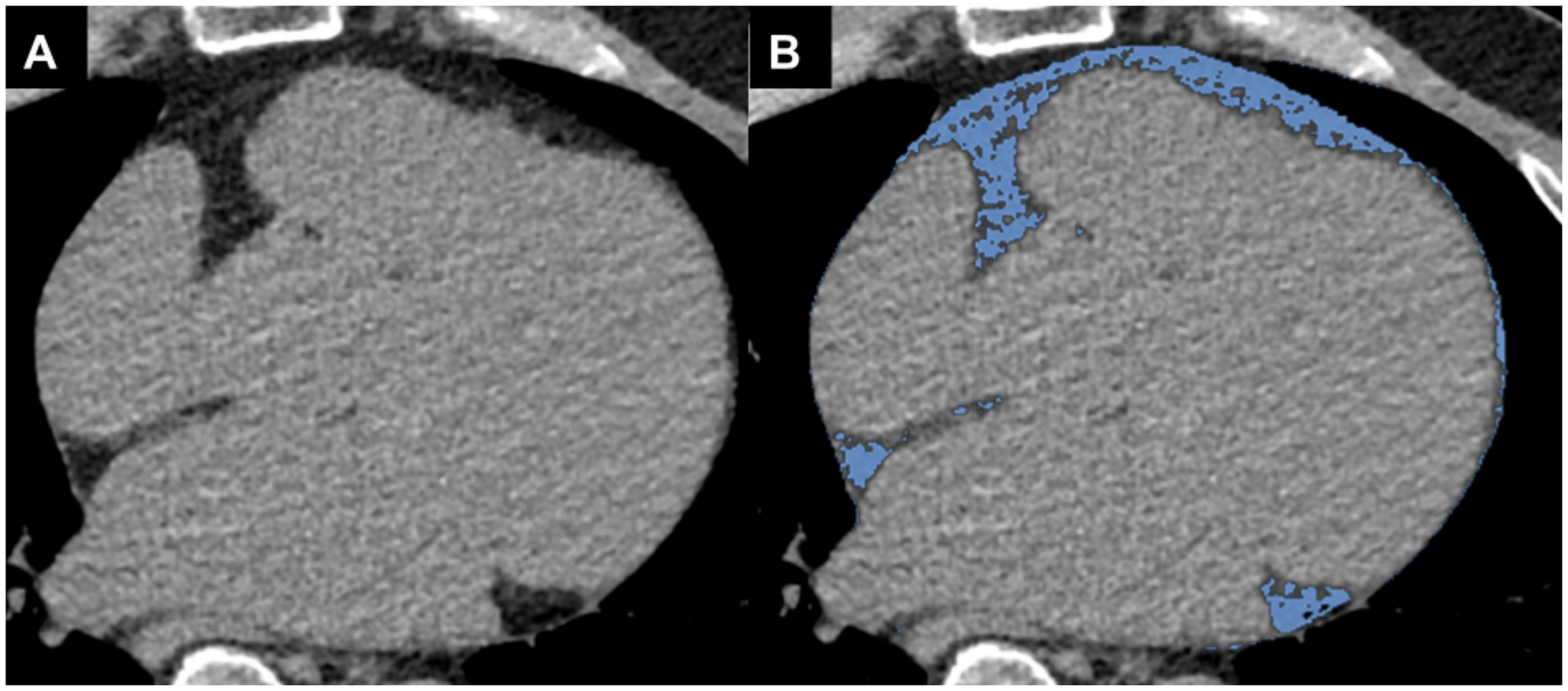
Epicardial adipose tissue (EAT) is segmented within the pericardial sac (HU range −190 to −30), providing volumetric and attenuation measures linked to systemic metabolic and inflammatory status. **(A)** Initial image showing the structure before segmentation. **(B)** Final segmented image focusing on the heart, achieved by applying a Hounsfield Unit (HU) threshold of −190 to −30 after initial segmentation, and obtained on Philips Software.

EAT volume and density are accurately quantified using CCTA. Volume is measured by segmenting the fat within a defined HU range (typically −190 to −30 HU) within the pericardial sac, extending from the pulmonary artery bifurcation to the apex of the left ventricle. Attenuation (radiodensity) of EAT, expressed in HU, serves as a surrogate marker of adipocyte composition and inflammation (68). Figure 5 demonstrates the stepwise segmentation of epicardial adipose tissue using standard HU thresholds. Lower attenuation reflects lipid-rich, poorly vascularized, and possibly inflamed or fibrotic “white” fat, whereas higher attenuation may indicate more thermogenically active or “brown-like” fat.

### Association with plaque burden, inflammation, and CAD risk

Increased EAT volume is independently linked to the presence and progression of CAD, coronary artery calcium (CAC), and MACE. In the Heinz Nixdorf Recall Study, higher EAT volume was associated with incident coronary heart disease after adjustment for age, sex, body mass index, and calcium score (16).

For EAT density, Goeller et al. reported that EAT attenuation below −75 HU was associated with a higher risk of myocardial infarction and cardiac death, and that EAT density showed a stronger independent association with MACE than EAT volume (68). In SMART, lower EAT density was associated with higher calcium burden in men with cardiovascular disease or high risk, independent of body mass index and EAT volume (69).

### Predictive role in subclinical atherosclerosis

EAT metrics capture subclinical atherosclerosis and cardiometabolic dysfunction. In a prospective cohort of heart failure with preserved ejection fraction (HFpEF) patients, lower EAT density was significantly associated with adverse cardiometabolic markers, including elevated fasting glucose, triglycerides, and insulin resistance scores, even after adjusting for body mass index and EAT volume (70). Lower EAT density was associated with risk of heart failure readmission, and improved EAT density was linked to heart failure with a higher incidence of heart failure with improved ejection fraction (HFimpEF) (70).

In the SCOT-HEART trial, EAT volume was independently associated with CAD, atrial fibrillation, all-cause mortality, myocardial infarction, and stroke, irrespective of traditional risk factors (*P* < 0.03) (71). Notably, EAT volume remained a significant predictor of all-cause mortality even after exclusion of cardiac deaths, suggesting a role in systemic metabolic dysfunction beyond its established association with coronary atherosclerosis (71).

## Pericoronary adipose tissue

### Distinction from epicardial adipose tissue

While EAT refers broadly to fat within the pericardial sac, PCAT specifically denotes the fat that immediately surrounds the coronary arteries. PCAT acts as a vascular niche, reflecting local inflammatory status due to its proximity to vessels and shared vasocrine signaling and embryological origin with vascular structures (72).

### Fat attenuation index and vessel-specific inflammation

PCAT attenuation, quantified in HU, shifts with local inflammation. Inflamed vessels release cytokines that inhibit lipid accumulation in PCAT, resulting in higher radiodensity (less negative HU values). The fat attenuation index (FAI) is calculated as the average attenuation of all voxels with HU values in the adipose tissue range (−190 to −30 HU) within a specified radial distance from the outer vessel wall, often equal to the vessel diameter (12).

Typical ranges include healthy PCAT at −90 to −70 HU, whereas inflamed vessels demonstrate FAI > −70 HU. The CRISP-CT study showed that FAI ≥ −70 HU around the proximal right coronary artery is associated with increased MACE and subclinical coronary inflammation (13). Extending these findings, the more recent ORFAN study, the largest outcome analysis to date comprising more than 40,000 patients, demonstrated that an elevated pericoronary FAI Score is associated with substantially higher cardiac mortality and MACE, particularly in patients without obstructive CAD, and improves risk reclassification when integrated with an artificial intelligence–based risk model (73). PCCT may also influence attenuation-based measurements of epicardial and PCAT, with potential downstream effects on the absolute thresholds used to define inflamed PCAT (74).

### Clinical correlates and therapeutic implications

Multiple meta-analyses and large cohort studies demonstrate that the pericoronary FAI independently predicts MACE after adjustment for conventional risk factors and CAC score (75, 76). Elevated FAI reflects coronary inflammation and identifies biologically active, high-risk plaques that are prone to rupture, thereby providing incremental prognostic information beyond stenosis severity or calcified burden (77).

The dynamic nature of FAI adds to its clinical value. Serial CCTA studies demonstrate that changes in FAI track longitudinal shifts in inflammatory activity and correlate with both plaque progression and clinical outcomes, supporting its potential role in dynamic risk monitoring (78). Furthermore, elevated FAI has been linked to impaired vascular reactivity, suggesting a mechanistic connection between pericoronary inflammation and endothelial dysfunction (79).

FAI shows particular promise in challenging clinical scenarios where traditional risk markers may be less discriminative. In high-risk populations such as patients with type 2 diabetes, lesion-specific FAI predicts MACE even in the presence of significant calcification, demonstrating its utility for refined risk stratification (80).

From a therapeutic perspective, FAI offers the potential to guide personalized prevention strategies by identifying individuals with residual inflammatory risk despite optimal lipid and blood pressure control (77, 81). This capability may prove especially valuable for informing the selective use of anti-inflammatory therapies, such as colchicine or IL-1β antagonists, in patients most likely to benefit, potentially serving as a companion diagnostic for the more costly agents in this class, such as IL-1β antagonists (82).

Evidence for FAI's responsiveness to treatment comes from statin studies, which show that high-dose statin therapy reduces FAI values in both noncalcified and mixed plaques (83–85). This reduction persists over time and occurs independently of low-density lipoprotein cholesterol (LDL-C) lowering, suggesting a pleiotropic anti-inflammatory effect that can be monitored through FAI measurements.

## Liver Fat and cardiometabolic risk

### CT-Based quantification of hepatic steatosis

Although hepatic steatosis is not a discrete adipose depot, it shares pathogenic links with visceral and pericoronary fat through common metabolic, inflammatory, and endocrine pathways (86). Noncontrast CT detects and quantifies liver fat via the inverse relationship between intrahepatic triglyceride content and attenuation. Normal liver tissue averages approximately 64 HU, with progressive decline as fat accumulates.

Two validated measurement approaches are used: (1) an absolute threshold of <48 HU, which indicates moderate-to-severe steatosis (≥30% fat content) with high specificity but limited sensitivity (87), and (2) the liver-to-spleen ratio (H/S ratio), where values <1.0 provide a standardized index less affected by scanner variations (88–90).

This approach is practical because it requires no additional radiation beyond the gated noncontrast CT for calcium scoring, which is frequently obtained alongside CCTA. AI-driven segmentation pipelines are making automated liver fat quantification increasingly accessible, with studies like EVAPORATE demonstrating excellent measurement reproducibility for longitudinal tracking (91). Recent data demonstrate that PCCT enables accurate liver fat quantification with excellent agreement to MRI-derived proton density fat fraction, which may overcome the sensitivity limitations of conventional noncontrast CT for mild steatosis.92.

Histologic fat fraction remains the diagnostic reference standard for hepatic steatosis, and correlative studies show that both CT attenuation and MRI proton density fat fraction (MRI-PDFF) track histologic fat content closely, with MRI-PDFF providing the most accurate and reproducible noninvasive quantification across the full spectrum of steatosis (87). On this basis, a pragmatic clinical algorithm can be proposed: hepatic steatosis is first screened opportunistically on the noncontrast calcium-score acquisition already obtained with CCTA (liver attenuation <48 HU or liver-to-spleen ratio <1.0); indeterminate or mild cases, in which CT sensitivity is limited, are referred for quantitative MRI-PDFF for accurate grading and longitudinal monitoring; and ultrasound-based vibration-controlled transient elastography with the controlled attenuation parameter serves as an accessible point-of-care alternative where MRI is unavailable or when concurrent assessment of hepatic fibrosis is required.

### Pathophysiologic mechanisms

Hepatic steatosis reflects systemic metabolic dysfunction and insulin resistance, and is closely linked to dyslipidemia and chronic inflammation (92, 93). Fat accumulation in the liver stimulates production of pro-inflammatory cytokines (TNF-α, IL-6), which contribute to endothelial dysfunction and atherosclerosis through both systemic and local vascular effects (94–97). The liver-heart axis encompasses multiple interconnected pathogenic mechanisms, including exacerbation of insulin resistance, genetic phenotypes, oxidative stress, atherogenic dyslipidemia, pro-inflammatory mediators, and alterations in gut microbiota (98). These inflammatory pathways mirror those observed in epicardial and pericoronary fat, suggesting shared pathophysiology between hepatic and cardiac fat depots (99).

### Clinical evidence and cardiovascular risk

Multiple large cohorts have established hepatic steatosis as an independent predictor of coronary disease. The Rotterdam Study demonstrated that lower hepatic attenuation correlated with increased CAC and epicardial fat, independent of traditional risk factors (100). The Framingham Heart Study showed that CT-detected fatty liver was associated with metabolic syndrome features even after adjusting for visceral adiposity (101). Additionally, the Paris Hepatology Atherosclerosis cohort found that fatty liver independently predicted carotid plaque, CAC, and multisite atherosclerosis with improved risk prediction beyond conventional factors (102).

Nonenhanced CT scans demonstrate that hepatic attenuation <40 HU is associated with increased volumes of noncalcified and low-attenuation plaque, features associated with vulnerability and adverse outcomes (103). Longitudinal studies (PARADIGM, ROMICAT-II) show that hepatic steatosis predicts both plaque progression over time and higher rates of high-risk plaque features and acute coronary syndromes (28, 104).

A comprehensive meta-analysis of over 34,000 adults found that imaging-defined NAFLD increased odds of combined fatal and non-fatal cardiovascular events by 64% (OR 1.64, 95% CI 1.26–2.13), with severe steatosis conferring even greater risk (OR ≈ 2.58) (105). Nested case-control studies demonstrate approximately 70% increased risk of major adverse cardiac events (MACE), independent of baseline coronary disease burden (103).

Recent data from spectral detector CT in diabetic patients showed that quantitative liver fat fraction significantly improved MACE prediction beyond traditional risk factors and coronary plaque metrics (106). The synergistic relationship between hepatic steatosis and high-risk coronary plaque features substantially elevates acute coronary event incidence.

### Clinical limitations and considerations

Noncontrast CT has notable limitations in assessing liver fat. The technique demonstrates only 57% sensitivity for mild steatosis (10%–20% fat content), while maintaining 88% specificity (87). Additionally, it cannot differentiate simple steatosis from steatohepatitis (nonalcoholic steatohepatitis [NASH]), and potential confounders such as iron overload, medications like amiodarone, glycogen storage, or contrast timing can affect interpretation.

NAFLD represents more than an incidental finding: it strongly correlates with insulin resistance and metabolic syndrome while independently increasing cardiovascular disease risk. Opportunistic liver fat quantification during noncontrast CT provides valuable metabolic risk information complementary to coronary assessment.

Incorporating hepatic fat measurements into cardiac CT workflows could improve personalized risk assessment. It is especially beneficial for patients with intermediate-risk profiles or those with inconsistent metabolic and lipid patterns. By utilizing existing imaging data, this method offers a thorough cardiometabolic evaluation without increasing radiation exposure or procedural complexity.

## Integrated imaging: multimarker strategy

### Rationale for combining multiple imaging biomarkers

**Figure 6:**
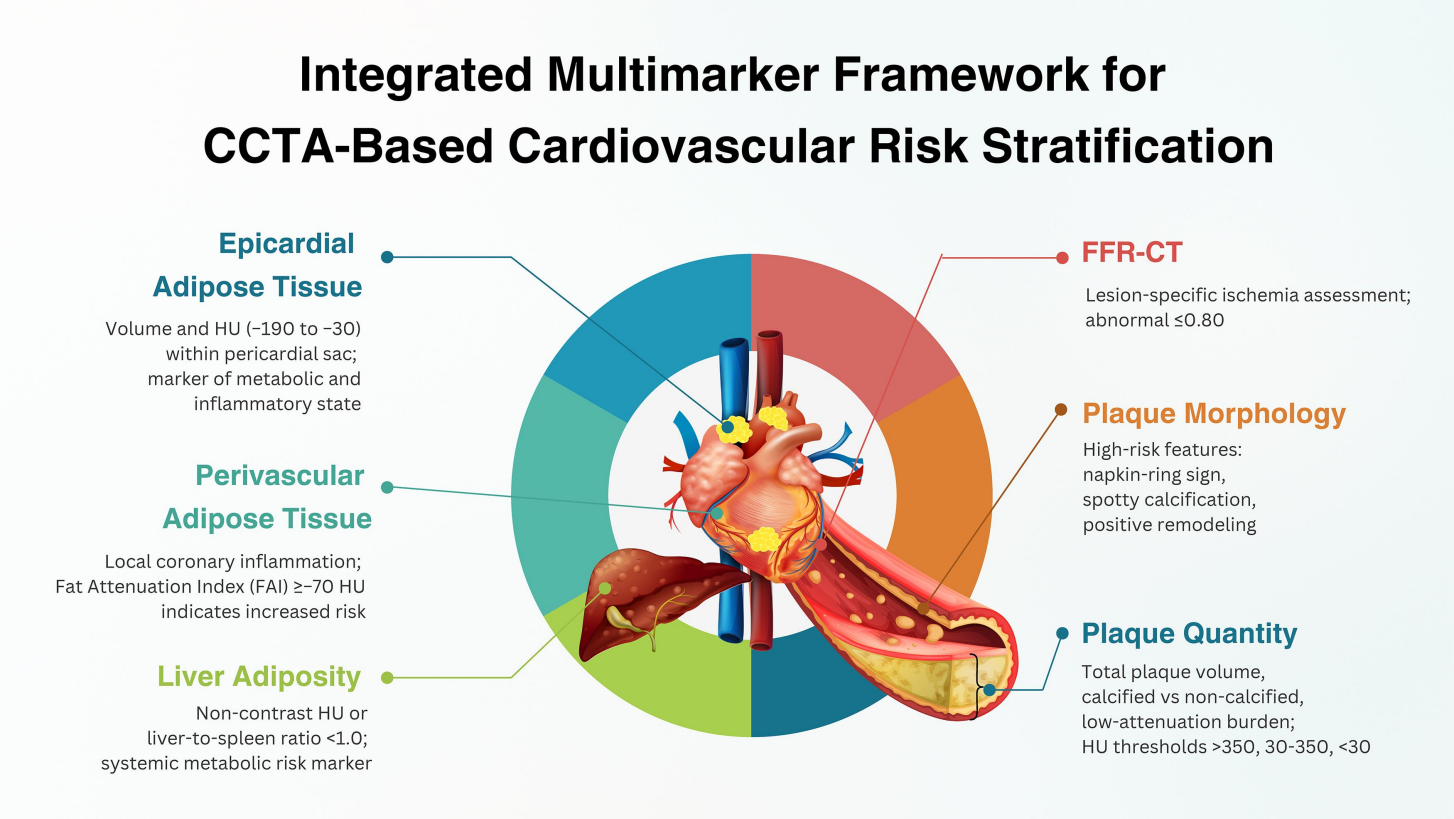
Integrated multimarker framework for CCTA-based cardiovascular risk stratification. Multiple quantitative imaging biomarkers are derived from a single CCTA scan, each reflecting a distinct pathobiologic domain: plaque quantity (anatomic burden), plaque morphology (high-risk structural features), FFR-CT (functional ischemia), epicardial adipose tissue (systemic inflammatory/metabolic status), pericoronary adipose tissue (local coronary inflammation), and liver adiposity (systemic metabolic risk). When combined, these metrics enable a multidimensional risk profile that improves the prediction of adverse cardiovascular events beyond stenosis severity alone.

The biology of CAD encompasses interrelated processes of plaque burden and vulnerability, hemodynamic significance, and vascular inflammation. No single imaging biomarker fully captures this spectrum, but CCTA uniquely allows integration across these domains. An integrated multimarker framework summarizing these complementary anatomic, functional, inflammatory, and metabolic domains is shown in Figure 6. Recent studies highlight the additive value of such a multimarker strategy: EAT volume improves discrimination of lesion-specific ischemia when combined with plaque quantification, with accuracy approaching that of FFR-CT (107); PCAT radiomics features further enhance detection of functionally relevant CAD beyond conventional stenosis and plaque measures (108); and in a single-center longitudinal study, EAT volume combined with plaque burden and FFR-CT independently predicted MACE with incremental discriminatory value beyond traditional clinical risk scores, a finding that requires confirmation in larger, multicenter cohorts (109).

Emerging data further support the prognostic superiority of composite over single-domain CCTA imaging biomarkers. Recent studies have shown that integrated scores combining plaque burden, high-risk plaque features, CAD-RADS classification, and pericoronary adipose tissue attenuation provide improved prognostic discrimination compared with any individual parameter (110). Consistent with this, evidence from the CONFIRM2 registry indicates that AI-driven quantitative CCTA analysis enhances cardiovascular risk discrimination beyond traditional metrics such as CAD-RADS and coronary artery calcium, reinforcing the rationale for a structured multimarker approach (111). An accompanying editorial commentary has likewise emphasized the complementary prognostic roles of plaque composition, epicardial adipose tissue, and pericoronary adipose tissue derived from cardiac CT (112). These reports, however, predominantly demonstrate gains in discrimination rather than improvements in calibration or clinically meaningful net reclassification; they are frequently single-center or only internally validated; and they have not shown that biomarker-guided management alters clinical decisions or improves patient outcomes. Prospective, externally validated studies with hard clinical endpoints are therefore needed before a composite multimarker strategy can be recommended for routine use.

More broadly, quantitative plaque assessment has become a foundational element of cardiovascular risk stratification, as AI-driven automated plaque measurements demonstrate enhanced predictive accuracy for cardiac events in multicenter patient cohorts (29, 113). Consensus statements have similarly emphasized the unique ability of CCTA to integrate plaque, flow, and fat as complementary markers of CAD risk (114). Collectively, these data support the hypothesis that a multimarker approach may refine ischemia prediction and improve prognostic discrimination; whether this translates into more personalized management or better patient outcomes remains to be demonstrated in prospective trials.

### Proposed clinical workflows

An integrated clinical workflow for CCTA could, in principle, sequentially combine anatomical, functional, and biological data to support risk stratification and management of CAD; we present the following as a conceptual, illustrative pathway rather than a validated or guideline-endorsed protocol. As illustrated in Figure 6, six distinct pathobiologic domains can be extracted from a single CCTA dataset: plaque quantity, reflecting overall anatomic burden; plaque morphology, capturing high-risk structural features such as low-attenuation and positively remodeled plaque; FFR-CT, providing lesion-specific functional ischemia assessment; epicardial adipose tissue, indexing systemic inflammatory and metabolic status; PCAT, reflecting local coronary inflammation; and liver adiposity, serving as a surrogate for systemic metabolic risk and insulin resistance. The evidence status of each domain and its supporting literature are summarized in Table 1. Plaque quantity carries Class I, LOE A recommendations in both the 2021 ACC/AHA and 2024 ESC guidelines and is a core component of CAD-RADS 2.0. Plaque morphology, including the high-risk plaque modifier, is incorporated into CAD-RADS 2.0 reporting and is recognized in the 2025 AHA Scientific Statement on nonobstructive CAD; however, it does not yet carry a standalone Class I recommendation in formal guideline documents (2, 3). FFR-CT occupies an intermediate position, carrying a Class 2a recommendation in the 2021 ACC/AHA Chest Pain Guideline for lesions with 40%–90% stenosis and incorporated as an ischemia modifier in CAD-RADS 2.0, though it does not yet hold a Class I recommendation for routine use (3, 4). The remaining three domains, EAT, PCAT, and liver adiposity, are investigational: major guidelines acknowledge their prognostic associations but do not recommend routine clinical measurement, citing the absence of standardized thresholds and limited evidence for treatment modification (1, 5, 9).

Semiautomated software then quantifies total plaque burden, composition, and vulnerability across the coronary tree, identifying at-risk patients even without obstructive disease and offering prognostic insights beyond stenosis severity alone (115).

FFR-CT is selectively applied to intermediate lesions to determine hemodynamic significance, reducing unnecessary invasive testing and guiding revascularization planning with support from SCCT and European Association of Cardiovascular Imaging consensus documents (3, 116, 117).

Epicardial and pericoronary adipose tissues are quantified using dedicated algorithms, capturing biological signals of vascular inflammation and cardiometabolic risk that provide incremental diagnostic value when combined with plaque metrics and FFR-CT (107, 109, 118). Opportunistic evaluation of extracardiac findings, including hepatic steatosis and aortic calcium, offers additional insights into systemic metabolic risk (114, 118).

Final reporting synthesizes plaque morphology, hemodynamic significance, and biological activity into a comprehensive risk profile that could inform preventive therapy, testing strategies, intervention planning, and identification of residual risk beyond traditional clinical factors (114, 115, 118). This stepwise workflow illustrates the emerging paradigm of CCTA as a multimarker platform; however, it is not yet supported by evidence that biomarker-guided management improves outcomes and should be regarded as a research framework pending prospective validation (3, 114, 116, 118).

## Limitations and challenges

### Technical challenges

Image quality and artifacts remain significant barriers to reliable analysis. Motion artifacts, suboptimal contrast timing, elevated heart rate, and heavy coronary calcification can all degrade scan interpretability, compromising the accuracy of low-attenuation plaque detection, FFR-CT computation, and segmentation of epicardial and PCAT (119).

Reproducibility is also challenged by variation in acquisition protocols across institutions. Differences in tube voltage, reconstruction kernels, and contrast protocols affect attenuation values and downstream quantitative metrics. The lack of standardized HU thresholds for defining plaque or fat characteristics, as well as variability in segmentation algorithms and inter-vendor software, limits cross-study comparisons and generalizability (120). Because these metrics are attenuation-based, low-attenuation plaque, EAT density, and FAI are particularly protocol-dependent and may not be directly comparable across scanners, tube voltages, reconstruction kernels, or software; published thresholds are therefore specific to the acquisition and analysis conditions in which they were derived, and this uncertainty should be carried into the interpretation of the supporting evidence. A practical overview of currently available software platforms across biomarker domains is provided in Supplementary Table S1.

### Clinical limitations

A significant clinical limitation is the lack of established cutoffs for many quantitative imaging biomarkers, such as EAT attenuation, pericoronary FAI, and liver HU values. Universally accepted clinical thresholds to guide treatment decisions and interventions do not yet exist, and these markers have not been incorporated into clinical practice guidelines or standard risk-stratification algorithms (121). This limits their routine clinical adoption. In addition, the adipose-tissue and hepatic imaging biomarkers (EAT, PCAT/FAI, and hepatic steatosis) are strongly influenced by obesity, diabetes, systemic inflammation, medication exposure, acquisition parameters, and population characteristics. Their reported associations with cardiovascular outcomes should therefore be regarded as predictive rather than causal in the absence of rigorous multivariable adjustment and longitudinal validation, and biological plausibility should not be equated with therapeutic actionability.

Beyond technical and analytical issues, real-world implementation is constrained by the accessibility and cost of the required software. Many of the imaging biomarkers reviewed here, including FFR-CT, advanced quantitative plaque analysis, and FAI, depend on proprietary, vendor-specific, or cloud-based platforms that involve substantial per-case or subscription costs, restricted geographic availability, and nontrivial integration with existing PACS and reporting workflows. These factors disproportionately affect community and lower-resource centers, contributing to inequitable access to advanced CCTA imaging biomarkers and slowing translation of guideline-supported tools such as FFR-CT into routine practice. Open, well-validated, and ideally on-site analytic solutions, together with transparent pricing and reimbursement frameworks, will be important enablers of broader clinical adoption (122). Workflow integration challenges also arise, as added layers of complexity can slow report turnaround times and impose workflow inefficiencies without robust automation. Several approaches also rely on proprietary or “black-box” AI whose training data and internal logic are not publicly available, raising additional concerns regarding external validation, algorithmic transparency and bias, regulatory clearance, data privacy, and reproducibility that compound the cost and access barriers noted above.

#### Research gaps

Significant gaps remain in the research on CT imaging biomarkers. A primary unmet need is longitudinal validation. While numerous studies have demonstrated cross-sectional associations between imaging biomarkers (such as FAI and plaque volume) and cardiovascular outcomes, few have investigated serial changes and their correlation with subsequent events. Most existing data are observational, and randomized trials employing biomarker-guided treatment strategies are currently lacking (84). This limitation is compounded by substantial underrepresentation of diverse populations within existing validation cohorts, where women, racial and ethnic minorities, and individuals with conditions such as HFpEF, chronic kidney disease, or inflammatory disorders are frequently excluded or insufficiently represented (123). In addition, head-to-head comparative studies across different commercial and academic software platforms applied to the same patient cohorts are urgently needed, as inter-vendor differences in segmentation algorithms, HU calibration, and AI training data may produce non-interchangeable values for plaque burden, FFR-CT, FAI, and EAT metrics, limiting cross-study comparability and clinical generalizability. Systematic comparison between conventional energy-integrating detector CT and PCCT in terms of biomarker quantification, reproducibility, and prognostic impact also represents an important and currently underexplored research priority. 112 Table 3 summarizes major technical, clinical, and research limitations affecting current quantitative and integrated CCTA imaging biomarkers.

**Table 3:** Current limitations and research gaps in quantitative and integrated CCTA imaging biomarkers.

Domain	Technical Limitations	Clinical Limitations	Research Needs
Quantitative Plaque Analysis	Variability in image acquisition, contrast timing, and reconstruction kernel; motion artifacts; inter-platform differences in HU thresholds and segmentation algorithms reduce reproducibility.	Lack of standardized cutoffs for low-attenuation plaque and non-calcified plaque burden; limited integration into guidelines.	Multicenter validation with standardized acquisition/analysis protocols; establish universally accepted risk thresholds; evaluate utility in diverse populations.
FFR-CT	Accuracy is dependent on image quality; heavy calcification and motion artifacts reduce reliability; high computational demands for CFD-based methods.	Limited validation in post-CABG, high CAC, and microvascular dysfunction; inconsistent reimbursement and cost barriers.	Broader validation in special populations; cost-effectiveness studies; refinement of machine learning-based, on-site processing models.
EAT	Segmentation variability; attenuation values influenced by acquisition parameters and tube voltage.	No consensus on normal vs. abnormal HU or volume thresholds; not yet incorporated into routine risk scores.	Define clinically actionable cutoffs; prospective trials assessing EAT-guided interventions; study sex and ethnic differences.
PCAT/FAI	HU measurements are sensitive to technical parameters; AI algorithms are not yet standardized across vendors.	No universally accepted FAI threshold; limited familiarity among clinicians.	Longitudinal studies linking FAI change to outcomes; randomized trials testing inflammation-targeted therapies guided by FAI.
Liver Fat Quantification	Non-contrast CT is less sensitive for mild steatosis; it cannot distinguish steatosis from steatohepatitis.	Not routinely quantified during CCTA; unclear treatment thresholds for cardiovascular risk modification.	Automated liver fat analysis integrated into CCTA workflows; define risk-based intervention thresholds; evaluate synergy with plaque and inflammatory markers.
Integrated Multimarker Models (anatomic + functional + inflammatory + metabolic CCTA imaging biomarkers combined)	Lack of standardized data fusion methods; inter-modality differences in measurement.	No validated composite risk score incorporating all imaging biomarkers; limited guideline endorsement.	Develop and validate integrated risk models; assess additive prognostic value in diverse, multi-ethnic cohorts; test biomarker-guided therapy strategies in randomized trials.

CCTA, coronary computed tomography angiography; FFR-CT, fractional flow reserve derived from CT; HU, Hounsfield units; FAI, fat attenuation index; EAT, epicardial adipose tissue; PCAT, pericoronary adipose tissue.

### Population diversity and biomarker expression

Population diversity critically affects biomarker expression and interpretation. Most validation studies have been conducted in predominantly White, male, and relatively low-risk populations, limiting generalizability across clinical practice. Sex-specific differences are increasingly recognized, as women often present with smaller coronary vessels, less calcified but more lipid-rich NCP, and distinct patterns of pericoronary fat inflammation that may affect both plaque quantification and FAI thresholds. Ethnic variation is equally significant: South Asian populations exhibit higher EAT volumes at lower body mass index, East Asian cohorts tend to have less calcified plaque but greater prevalence of positive remodeling, and African American populations may demonstrate higher pericoronary fat attenuation despite similar plaque burden, suggesting differences in inflammatory phenotype. Patients with metabolic syndrome or type 2 diabetes display disproportionately high liver fat and PCAT inflammation, often without parallel increases in CAC, underscoring the potential value of metabolic biomarkers in these high-risk groups.

Without targeted recruitment of women, racially diverse cohorts, and populations with metabolic comorbidities into biomarker validation trials, risk prediction models may fail to capture these population-specific nuances, potentially perpetuating health disparities in cardiovascular prevention. Future research must prioritize longitudinal studies across diverse populations to establish population-specific thresholds and validate biomarker-guided treatment strategies that ensure equitable application of quantitative CCTA tools. In particular, universal thresholds derived largely from these cohorts (for example, FAI ≥ −70 HU, EAT density cutoffs, and hepatic HU or liver-to-spleen ratios) may not transfer across sexes, ethnicities, body-composition strata, or comorbid populations, and should be applied cautiously until validated in the relevant group.

## Future perspectives

### Artificial intelligence and deep learning

AI-driven image analysis is rapidly transforming the interpretation of CCTA. Convolutional neural networks (CNNs) now enable fully automated plaque segmentation, volumetric quantification, and compositional analysis with high reproducibility and near–real-time performance. Deep learning–based alternatives to CFD have also accelerated the adoption of FFR-CT by significantly reducing processing time, enabling functional lesion assessment directly within clinical workflows (45, 46). On-site FFR-CT solutions, which run locally on hospital workstations rather than relying on cloud-based services, further shorten turnaround time, reduce dependence on third-party platforms, and may improve integration with same-day diagnostic and treatment pathways (41, 124).

To mitigate the interplatform variability in fixed HU thresholds noted earlier, adaptive thresholding strategies that normalize plaque attenuation to scan-specific luminal contrast intensity have been proposed and may improve reproducibility of compositional plaque metrics across scanners and protocols (125).

Pericoronary inflammation imaging has advanced through radiotranscriptomic models that infer gene expression signatures from CT-derived PCAT features, such as the FAI. These models provide a mechanistic link between imaging phenotypes and vascular biology, enhancing risk prediction beyond conventional metrics (126).

Building on these developments, AI-based platforms are being designed to integrate CCTA imaging biomarkers with clinical, genomic, proteomic, and metabolomic data to generate individualized cardiovascular risk scores. Early proof-of-concept studies suggest that multi-omics fusion with imaging phenotypes may improve early identification of high-risk individuals and aid in the discovery of novel therapeutic targets. These integrative frameworks could mark a transition from population-based risk models to truly personalized cardiovascular medicine (127).

### Emerging CT technologies

PCCT and spectral/dual-energy CT are poised to substantially refine quantitative CCTA imaging biomarkers. PCCT enables higher spatial resolution, reduced partial volume averaging, and improved plaque component differentiation (128). Spectral CT additionally allows virtual monoenergetic and material-specific reconstructions that may stabilize HU-based metrics across acquisition parameters. Beyond the coronary tree, CT-derived myocardial extracellular volume (ECV) provides a noninvasive surrogate of diffuse interstitial fibrosis with high prognostic value for cardiovascular events, and can be obtained from the same delayed-phase acquisition used for CCTA (129). Systematic comparison of conventional CT and PCCT for biomarker quantification, reproducibility, and prognostic impact represents an important and currently underexplored research priority.

### Longitudinal imaging and disease monitoring

Serial CCTA is increasingly being leveraged not merely for diagnosis but as a dynamic modality for monitoring disease progression and therapeutic response. Noninvasive serial imaging allows quantification of plaque progression or regression, assessment of changes in pericoronary and epicardial fat inflammation, and evaluation of high-risk plaque morphology over time (130). Routine serial CCTA for imaging biomarker monitoring nonetheless entails repeated ionizing-radiation and iodinated-contrast exposure, additional cost, and potential downstream testing, and its incremental value over standard care has not been established; broad clinical use should await prospective evidence that serial imaging-guided management improves outcomes.

Future clinical trials are expected to adopt serial imaging endpoints, such as quantification of LAP volume and dynamic tracking of pericoronary FAI, to evaluate pharmacologic efficacy beyond conventional metrics like stenosis degree or lumen diameter.

### Personalized and preventive cardiology

Individualized treatment strategies can be guided by combining anatomic stenosis with high-risk plaque features, functional ischemia assessment via FFR-CT, evaluation of the inflammatory state via PCAT FAI and EAT, and systemic metabolic risk assessment via hepatic steatosis (126). Cloud-based AI platforms could make advanced analysis accessible even in nontertiary centers. Integrating structured CCTA biomarker reporting into electronic medical records and decision-support tools will improve real-time clinical utility (131).

## Conclusion

CCTA has evolved far beyond its traditional role in evaluating coronary stenosis. Advances in postprocessing techniques and AI have transformed CCTA into a multimodal platform that integrates anatomic, functional, inflammatory, and metabolic insights from a single scan.

This review highlights the growing clinical relevance of several quantitative imaging biomarkers. Plaque characteristics, including low-attenuation, noncalcified, and positively remodeled plaque, provide early indicators of lesion vulnerability. FFR-CT offers lesion-specific physiological assessment and reduces reliance on invasive procedures. Epicardial and pericoronary fat serve as surrogates for local and systemic inflammation, while liver fat reflects cardiometabolic stress and insulin resistance.

When considered together, these imaging biomarkers provide a layered risk profile that more accurately captures the complex interplay among coronary anatomy, hemodynamics, inflammation, and metabolic dysfunction. Such a multidimensional assessment may improve prognostic accuracy, although to date this has been shown chiefly as incremental discrimination in observational studies rather than as improved patient outcomes. This framework is best regarded as a hypothesis-generating model that may inform future individualized risk assessment and preventive strategies once prospectively validated.

While several challenges remain, including standardization, accessibility, and longitudinal validation, the field advances rapidly. Emerging AI tools, radiotranscriptomic techniques, and integration with omics data will further refine biomarker extraction, automate interpretation, and support clinical decision-making.

CCTA is transitioning from a gatekeeper for invasive angiography to a proactive tool for prediction and prevention. With standardization and prospective, outcome-based validation, the incorporation of emerging quantitative imaging biomarkers has the potential to refine cardiovascular risk stratification and support targeted prevention; realizing this potential will require rigorous, adequately powered studies demonstrating that biomarker-guided care improves patient outcomes.
