# Supplementary material for: Emerging quantitative CCTA imaging biomarkers for cardiovascular risk stratification: a narrative review
Source: Front Cardiovasc Med. 2026 Aug 4;13:1850945. doi: 10.3389/fcvm.2026.1850945 (PMC13482244; doi:10.3389/fcvm.2026.1850945)
Supplement: Supplementary file 1 [file Table1.docx]

## Reference

[52.](https://www.zotero.org/google-docs/?SbltKB)	[Varga-Szemes A, Schoepf UJ, Maurovich-Horvat P, et al. Coronary plaque assessment of Vasodilative capacity by CT angiography effectively estimates fractional flow reserve. *Int J Cardiol*. 2021;331:307-315. doi:10.1016/j.ijcard.2021.01.040](https://www.zotero.org/google-docs/?SbltKB)

[132.](https://www.zotero.org/google-docs/?SbltKB)	[Fairbairn TA, Rodriguez F, Martin S, et al. Modelling the long-term clinical outcomes of AI-enabled CCTA plaque quantification on preventive cardiovascular management. *Eur J Prev Cardiol*. 2025;32(Supplement_1):zwaf236.001. doi:10.1093/eurjpc/zwaf236.001](https://www.zotero.org/google-docs/?SbltKB)

[133.](https://www.zotero.org/google-docs/?SbltKB)	[Dahdal J, Jukema RA, Maaniitty T, et al. CCTA-Derived coronary plaque burden offers enhanced prognostic value over CAC scoring in suspected CAD patients. *Eur Heart J - Cardiovasc Imaging*. 2025;26(6):945-954. doi:10.1093/ehjci/jeaf093](https://www.zotero.org/google-docs/?SbltKB)

[134.](https://www.zotero.org/google-docs/?SbltKB)	[Lee SN, Lin A, Dey D, Berman DS, Han D. Application of Quantitative Assessment of Coronary Atherosclerosis by Coronary Computed Tomographic Angiography. *Korean J Radiol*. 2024;25(6):518. doi:10.3348/kjr.2023.1311](https://www.zotero.org/google-docs/?SbltKB)

[135.](https://www.zotero.org/google-docs/?SbltKB)	[Park HB, Lee BK, Shin S, et al. Clinical Feasibility of 3D Automated Coronary Atherosclerotic Plaque Quantification Algorithm on Coronary Computed Tomography Angiography: Comparison with Intravascular Ultrasound. *Eur Radiol*. 2015;25(10):3073-3083. doi:10.1007/s00330-015-3698-z](https://www.zotero.org/google-docs/?SbltKB)

[136.](https://www.zotero.org/google-docs/?SbltKB)	[Weichsel L, Giesen A, André F, et al. Comparison of Two Contemporary Quantitative Atherosclerotic Plaque Assessment Tools for Coronary Computed Tomography Angiography: Single-Center Analysis and Multi-Center Patient Cohort Validation. *Diagnostics*. 2024;14(2):154. doi:10.3390/diagnostics14020154](https://www.zotero.org/google-docs/?SbltKB)

[137.](https://www.zotero.org/google-docs/?SbltKB)	[Michail M, Ihdayhid AR, Comella A, et al. Feasibility and Validity of Computed Tomography-Derived Fractional Flow Reserve in Patients With Severe Aortic Stenosis: The CAST-FFR Study. *Circ Cardiovasc Interv*. 2021;14(1):e009586. doi:10.1161/CIRCINTERVENTIONS.120.009586](https://www.zotero.org/google-docs/?SbltKB)

[138.](https://www.zotero.org/google-docs/?SbltKB)	[Tjellaug Braaten A, Fossan FE, Muller L, et al. An automated reduced order CT-FFR model for diagnosis of hemodynamically significant coronary artery disease- a prospective validation study. *Eur Heart J*. 2023;44(Supplement_2):ehad655.158. doi:10.1093/eurheartj/ehad655.158](https://www.zotero.org/google-docs/?SbltKB)

[139.](https://www.zotero.org/google-docs/?SbltKB)	[Bråten AT, Fossan FE, Muller LO, et al. Automated computed tomography-derived fractional flow reserve model for diagnosing haemodynamically significant coronary artery disease: a prospective validation study. *Eur Heart J - Imaging Methods Pract*. 2024;2(3):qyae102. doi:10.1093/ehjimp/qyae102](https://www.zotero.org/google-docs/?SbltKB)

[140.](https://www.zotero.org/google-docs/?SbltKB)	[Coenen A, Kim YH, Kruk M, et al. Diagnostic Accuracy of a Machine-Learning Approach to Coronary Computed Tomographic Angiography–Based Fractional Flow Reserve: Result From the MACHINE Consortium. *Circ Cardiovasc Imaging*. 2018;11(6):e007217. doi:10.1161/CIRCIMAGING.117.007217](https://www.zotero.org/google-docs/?SbltKB)

[141.](https://www.zotero.org/google-docs/?SbltKB)	[Nous FMA, Budde RPJ, Lubbers MM, et al. Impact of machine-learning CT-derived fractional flow reserve for the diagnosis and management of coronary artery disease in the randomized CRESCENT trials. *Eur Radiol*. 2020;30(7):3692-3701. doi:10.1007/s00330-020-06778-w](https://www.zotero.org/google-docs/?SbltKB)

[142.](https://www.zotero.org/google-docs/?SbltKB)	[Coenen A, Lubbers MM, Kurata A, et al. Fractional Flow Reserve Computed from Noninvasive CT Angiography Data: Diagnostic Performance of an On-Site Clinician-operated Computational Fluid Dynamics Algorithm. *Radiology*. 2015;274(3):674-683. doi:10.1148/radiol.14140992](https://www.zotero.org/google-docs/?SbltKB)

[143.](https://www.zotero.org/google-docs/?SbltKB)	[Bozika M, Apostolos A, Nastouli KM, et al. Clinical Impact of CT-Based FFR in Everyday Cardiology: Bridging Computation and Decision-Making. *Biomedicines*. 2025;13(8):1969. doi:10.3390/biomedicines13081969](https://www.zotero.org/google-docs/?SbltKB)

[144.](https://www.zotero.org/google-docs/?SbltKB)	[Yang J, Shan D, Wang X, et al. On-Site Computed Tomography–Derived Fractional Flow Reserve to Guide Management of Patients With Stable Coronary Artery Disease: The TARGET Randomized Trial. *Circulation*. 2023;147(18):1369-1381. doi:10.1161/CIRCULATIONAHA.123.063996](https://www.zotero.org/google-docs/?SbltKB)

**Supplementary Table S1.** Currently available software platforms for CCTA-derived imaging biomarkers, with deployment model and representative validation studies

| **Biomarker Domain** | **Platform** | **Vendor** | **Platform Type** | **Deployment** | **Representative Validation** |
| --- | --- | --- | --- | --- | --- |
| **Quantitative Plaque Analysis** | AutoPlaque (v2.5) | Cedars-Sinai Medical Center | Semi-automated; academic | On-site | Semi-automated quantitative plaque analysis used as reference standard in an international multicentre study; strong agreement with deep-learning system and IVUS; expert AutoPlaque measurements prognostic for MI in SCOT-HEART.[^54^](https://www.zotero.org/google-docs/?7zZtp3) |
|  | AI-QCPA / AI-QP  (HeartFlow Plaque Analysis) | HeartFlow, Inc. | AI-based; commercial | Cloud | REVEALPLAQUE: strong correlation vs IVUS for total, calcified, and non-calcified plaque volumes (n=237 pts, 432 lesions)[^24^](https://www.zotero.org/google-docs/?yx2yI1)  ISCHEMIA: plaque burden independently predicted CV death/MI beyond risk factors[^23^](https://www.zotero.org/google-docs/?EvOwPn)  FISH&CHIPS: AI-QP-guided strategy projected 10-year risk reduction vs standard CCTA reporting.[^132^](https://www.zotero.org/google-docs/?QtE6Ph) |
|  | Cleerly AI-QCT  Plaque Analysis | Cleerly, Inc. | AI-based; commercial | Cloud | 2,404 pts with suspected CAD: AI plaque metrics (PAV, NCPV%) outperformed CAC for prediction of death/MI at 7 years; NCPV% highest AUC for MI.[^133^](https://www.zotero.org/google-docs/?t7cyAa)  PARADIGM substudies: serial plaque quantification with reduced measurement variability.[^134^](https://www.zotero.org/google-docs/?x3duev) |
|  | QAngio CT / QCT | Medis Medical Imaging | Semi-automated; commercial | On-site | Validated vs IVUS for lumen area, stenosis, and plaque burden.  PARADIGM registry (>2,000 pts, ≥2-year serial CCTA): plaque subtypes, progression, and statin effects quantified.[^134,135^](https://www.zotero.org/google-docs/?ujJ5ow) |
|  | syngo.via Frontier  (CT Coronary Plaque Analysis) | Siemens Healthineers | AI-assisted; commercial | On-site | Multicentre cohort (100 pts, 300 vessels): automated DL tool showed markedly lower inter/intra-observer variability (~2–4% vs ~20–26%) and ~50% shorter analysis time vs manual.[^136^](https://www.zotero.org/google-docs/?lqL6j2) |
|  | SUREplaque | Canon Medical Systems | Semi-automated; commercial | On-site | Prospective validation vs IVUS with radiofrequency backscatter; low-density NCP correlated with necrotic core/fibrofatty tissue on IVUS.[^134^](https://www.zotero.org/google-docs/?usccJW) |
|  | vascuCAP / PlaqueID  (Elucid Bioimaging) | Elucid, Inc. | AI-based; commercial | On-site / Cloud | Validated vs carotid CT histopathology for calcification and lipid-rich necrotic core.  EVAPORATE RCT: coronary plaque morphology changes tracked at 9 and 18 months under icosapent ethyl therapy.[^134^](https://www.zotero.org/google-docs/?Swe77L) |
| **FFR-CT** | HeartFlow FFR-CT  (FFRCT) | HeartFlow, Inc. | AI/CFD-based; commercial | Cloud | Used as core-lab CT-FFR in severe aortic stenosis (CAST-FFR), showing good correlation with invasive FFR and 76.7% diagnostic accuracy in 60 vessels.[^137^](https://www.zotero.org/google-docs/?UmXBFo) |
|  | Siemens cFFR  (on-site CFD) | Siemens Healthineers | CFD-based; commercial | On-site | Prospective validation (108 pts): AUC 0.85 vs invasive FFR, specificity 84%.[^138,139^](https://www.zotero.org/google-docs/?xN6YgJ)  MACHINE consortium: ML CT-FFR matched CFD CT-FFR (AUC 0.84) and outperformed visual CCTA in 351 pts.[^140^](https://www.zotero.org/google-docs/?w0uLpN)  CRESCENT trials: on-site CT-FFR would have reduced additional testing and unnecessary ICA.[^141^](https://www.zotero.org/google-docs/?Zky59i)  On-site CFD (106 pts, 189 vessels): AUC 0.83 vs invasive FFR, better accuracy than CCTA alone.[^142^](https://www.zotero.org/google-docs/?ydUYG4) |
|  | DEEPVESSEL FFR /  DL FFR-CT | Keya Medical / Academic | AI/DL-based; commercial/academic | On-site / Cloud | ADAPT multicentre observational study (269 pts, 358 vessels): validated vs invasive FFR; platform has CE, NMPA, FDA, and HSA approvals.[^143^](https://www.zotero.org/google-docs/?Qo98Z3)  TARGET RCT (1,216 pts): on-site DEEPVESSEL CT-FFR care pathway reduced ICA without obstructive CAD but showed no 1-year MACE or QoL benefit.[^144^](https://www.zotero.org/google-docs/?leKTP6) |

*Listing is comprehensive but not exhaustive and reflects platforms with peer-reviewed clinical outcome or reference-standard validation data. Inclusion does not imply endorsement; absence does not imply unsuitability. Deployment indicates the predominant operational model in current or emerging clinical use. Vendor information is provided for orientation only and may change as the regulatory and commercial landscape evolves. AI = artificial intelligence; AUC = area under the curve; CAC = coronary artery calcium; CCTA = coronary computed tomography angiography; CFD = computational fluid dynamics; DL = deep learning; FFR-CT = fractional flow reserve derived from coronary CT angiography; HRP = high-risk plaque; IVUS = intravascular ultrasound; MACE = major adverse cardiovascular events; MRI-PDFF = MRI proton density fat fraction; NCP = noncalcified plaque; NCPV = noncalcified plaque volume; PAV = percent atheroma volume; PCCT = photon-counting CT; PCAT = pericoronary adipose tissue; SCCT = Society of Cardiovascular Computed Tomography.*
